# Prevalence of antibody to *Trypanosoma cruzi *in Hispanic-surnamed patients seen at Parkland Health & Hospital System, Dallas, Texas

**DOI:** 10.1186/1756-0500-4-132

**Published:** 2011-04-29

**Authors:** Roberto Arena, Christine E Mathews, Anne Y Kim, Tim E Lenz, Paul M Southern

**Affiliations:** 1 Department of Pathology, University of Texas Southwestern Medical Center, Dallas, Texas, USA; 2 Department of Pathology, Parkland Hospital & Health System, Dallas, Texas, USA

## Abstract

**Background:**

Chagas disease constitutes an important public health threat in terms of morbidity and mortality in the areas in the United States where immigrant populations from Latin America are conspicuous. We conducted a survey to assess the prevalence of anti-*T. cruzi *antibody in Hispanic-surnamed patients seen at Parkland Memorial Hospital in Dallas, Texas.

**Findings:**

Five hundred serum specimens from Hispanic-surnamed patients were tested by a preliminary ELISA method. On a subset of 50 sera confirmatory testing was also performed using an alternative ELISA, indirect immunofluorescence, and TESA immunoblot. For 274 of 500 Hispanic-surnamed patients, we were able to ascertain immigration status upon medical chart review. Of the 274 sera analyzed, one sample tested as positive for anti-*T. cruzi *antibody by the preliminary ELISA, and by the three confirmatory methods.

**Conclusions:**

The goal of this study is to increase the awareness of *T. cruzi *infection and Chagas disease in areas where the Latin American immigrant communities are growing. Our study highlights the importance of testing for Chagas disease in the populations most at risk, and the need for current data on the actual seroprevalence in areas where such immigrant populations are conspicuous. Larger-scale epidemiologic surveys on Chagas disease in the immigrant communities from Latin America are warranted.

## Background

Chagas disease, also known as American trypanosomiasis, is an infection caused by the parasite *Trypanosoma cruzi*. This parasite is usually transmitted to humans by Triatominae insects as vectors. Transmission of the infection also occurs transplacentally, via blood transfusion, organ transplantation, laboratory incident, and ingestion of triatomine-contaminated food or drink [1-12]. Chagas disease has an acute stage, typically asymptomatic or with mild symptoms (e.g., fever, malaise, swelling at the site of inoculation, and lymphadenopathy) during the first 6 to 8 weeks after infection. This acute stage is often undetected and thus not treated. If not treated, Chagas disease becomes a chronic, lifelong condition which can go undetected for several decades in any given patient. The majority of infected persons remain asymptomatic in the chronic indeterminate phase (i.e., a prolonged period of clinically silent infection that follows acute primary infection). However, an estimated ~30% of infected individuals will have onset of chronic symptomatic disease, usually decades after the initial infection, with cardiac manifestations (e.g., cardiomyopathy, arrhythmias, and sudden death) or gastrointestinal involvement (e.g., megaesophagus and megacolon) [2,13]. Currently available drugs for Chagas disease require a prolonged course, pose a significant risk of adverse effects, and require careful monitoring [14]. Chagas disease is a major public health concern in Latin America where 8-10 million persons are chronically infected [12]. Historically, it has been considered as endemic mostly in the rural areas of Latin America [12], and the prevalence rate of *T. cruzi *infection in the endemic areas varies considerably from country to country in Mexico, Central and South America. The epidemiology of Chagas disease has been greatly modified in recent times by urbanization and international migration [15-20]. Unrecognized disease in the immigrant populations might lead to emerging health problems within the areas in the United States where immigrant communities are growing. Prior infection with *T. cruzi *has been previously documented in such populations by blood donor screening and follow up [7,8,10-12]. Estimates of *T. cruzi *antibody prevalence in the blood donor population in the United States vary widely, reflecting geographic differences as well as a trend of steady increase in prevalence as a result of conspicuous immigration from the endemic areas of Latin America over the last two decades [7,8,10-12]. In a recent survey by Tobler et al.[11], the seroprevalence was found to be 0.03% among blood donations in a region where 78% of the residents were of Hispanic origin. However, any volunteer blood donor population represents a highly selected group due to the selection bias introduced by the "healthy blood donor effect", and the corresponding seroprevalence rates are expected to underestimate the true prevalence rate of anti-*T. cruzi *antibody in the Latin American immigrant population. We conducted a survey to assess the prevalence of anti-*T. cruzi *antibody in Hispanic-surnamed patients seen at Parkland Memorial Hospital in Dallas, Texas. The Dallas-Fort Worth metroplex area includes a large immigrant population from Latin America. As the county hospital for Dallas, Texas, Parkland Health & Hospital System serves the local Hispanic communities, and the immigrant community originating from Mexico represents a significant proportion of our patient population.

## Methods

### Study population

We obtained the serum samples from 500 Hispanic-surnamed patients seen at Parkland Memorial Hospital between November 2008 and May 2009. Specifically, we used left-over serum samples collected from 500 Hispanic-surnamed in-patients or out-patients that were sent for testing to the immunology laboratory at Parkland Memorial Hospital. That is, from serum samples collected for diagnostic or treatment purposes (e.g., hepatitis serology or other infectious disease serology) and only after such testing for diagnostic and treatment purposes had been performed. For each of the 500 Hispanic-surnamed patients from which a serum sample was obtained, the electronic medical record was reviewed, and any evidence of immigration status from Latin America was noted. Specifically, we searched the medical record for notations such as "Immigrant from Latin America" or "Spanish-speaking only" as evidence of their immigration status from Latin America. This is an incomplete index of the immigrant status of our patient population because it is not the hospital's policy to enquire regarding this issue.

### Ethical consideration

The study was reviewed and approved by the Institutional Review Board of University of Texas Southwestern and by the Institutional Review Board of Parkland Memorial Hospital. A waiver of informed consent of the subjects included in the study was obtained from the IRB for the purpose of this epidemiologic survey. Left-overs from serum samples collected solely for non-research purposes such as diagnosis and treatment were used in this study. In the clinical immunology laboratory, upon selection of the Latin American-surnamed patients to include in the study, the left-over serum sample from such patients was transferred to a new tube which was not labeled with patient identifiers. A non-identifiable code was assigned to each left-over sample obtained for the study so that no direct link to a specific patient was possible.

### Serologic testing for Chagas disease

Parkland Memorial Hospital clinical immunology laboratory was requested to save all left-over serum specimens collected for diagnostic and/or treatment purposes from in-patients and out-patients from the date of IRB approval until May 2009. The serum was transferred to a new tube and was stored at - 70 C until use.

#### Enzyme-linked immunosorbent assay

Enzyme-linked immunosorbent assay (Hemagen Chagas' Kit - EIA Method; Hemagen Diagnostics, Inc, Columbia, MD) was carried out according to the manufacturer's protocol on all 500 serum samples included in the study. Absorbance was measured spectrophotometrically at 450 nm. Positive and negative controls were included in duplicate in each run, and yielded the expected results.

#### Confirmatory testing for Chagas disease

Of the 500 serum samples included in the study and tested by EIA method using the Hemagen Chagas' kit as discussed above, confirmatory testing was carried out on a subset of 50 sera (see Results for selection criteria) at the Centers for Disease Control and Prevention (CDC) in the Parasitic Diseases Branch Laboratory. The confirmatory assays used were Chagatest - ELISA Recombinante v 3.0 (Wiener Laboratorios, Rosario, Argentina), indirect immunofluorescence testing (IIF) [21], and finally an Immunoblot assay using trypomastigote excreted-secreted antigens (TESA-blot) [22]. For each of the three confirmatory assays, positive and negative controls were included in duplicate, and yielded the expected results. The TESA-blot is performed using excreted-secreted antigens that are spontaneously released by the trypomastigote form into the culture medium, and used in an immunoblot format for the detection of anti-*T. cruzi *antibody in infected individuals. Briefly, the TESA immunoblot technique is a solid-phase immunoassay in which the trypomastigote excreted-secreted antigens ("TESA") are separated using gel electrophoresis and then transferred to a nitrocellulose membrane. The anti-*T. cruzi *antibody from the serum of an infected patient will bind to the immobilized antigen, and the antibody-antigen reaction is detected exploiting an EIA-like enzymatic reaction that yields a colored end-product.

## Results

We initially tested 500 serum specimens from Hispanic-surnamed patients seen at Parkland using the Hemagen ELISA kit. Of these 500 sera, 428 were obtained from men, and 72 were obtained from women. Age ranged from 13 to 92 years, with a median age of 44 years. Of the 500 sera, one sample tested as positive for anti-*T. cruzi *antibody by Hemagen ELISA. For 274 of 500 Hispanic-surnamed patients, we were able to ascertain immigration status upon medical chart review. Specifically the electronic medical record of these 274 Hispanic-surnamed patients contained evidence of immigration status from Latin America. Of these 274 sera, 231 were obtained from men, 43 were obtained from women, and median age was 47 years. This set of 274 sera included the one sample that tested as positive for anti-*T. cruzi *antibody by Hemagen ELISA, as reported above. For the remaining 226 of these 500 subjects, upon chart review, there was no evidence regarding immigration status from Latin America. None of these 226 tested as positive for anti-*T. cruzi *antibody by Hemagen ELISA. We excluded from our analysis results from the 226 patients for whom no evidence was found regarding their immigration status. The subset of 50 samples sent to CDC for confirmatory testing included the one sample that tested as positive by Hemagen, along with other 49 randomly selected samples. The confirmatory testing on this preliminary positive serum yielded a positive result also using the Wiener recombinant EIA method, as well as by IIF and TESA immunoblot. Of the remaining 49 randomly selected serum samples sent to CDC for confirmatory testing, one additional sample yielded a result close to the cutoff value for the ELISA Wiener Recombinante (OD value 0.453, cutoff OD level 0.40) but negative on the other two tests, IIF and TESA immunoblot. According to the WHO guidelines for the laboratory diagnosis of Chagas disease [1], different algorithms should be used for the diagnosis of *T. cruzi *infection depending on the specific purpose for laboratory testing. That is, two tests should be used to confirm a clinical suspicion of Chagas disease in a given patient, and a third test should be performed if the results of the two tests are not concordant. For the purpose of an epidemiologic survey or for blood donor screening, a single test can be used, given the excellent sensitivity of currently available assays. In this survey, given the availability at the CDC of a variety of assays for confirmatory testing, seropositivity results from the concordant positive result of all the four testing methodologies employed, exceeding even the most stringent WHO criteria and testing algorithm aimed at maximizing the specificity for the clinical diagnosis of Chagas disease. Our survey resulted in a seroprevalence of 0.4% (1/274) (95% CI 0%, 1.08%) among Hispanic-surnamed patients selected for inclusion in this survey analysis.

## Discussion

The estimated 0.4% seroprevalence is derived from testing of a convenience sample of left-over serum samples from patients who had diagnostic testing done for purposes unrelated to Chagas disease. A drawback of this study is given by the limited subset of samples on which the additional confirmatory testing was performed. Another limitation of this study is possible misclassification of immigration status for individuals who were truly immigrants but did not have that noted in their medical records. However, 0.4% seroprevalence is comparable to the estimated prevalence of Chagas disease in some areas of Mexico, based on blood donor screening results, and the testing criteria for seropositivity in this study were more rigorous than most [1,23]. Given the natural history of Chagas disease and the limitations of currently available treatments, early detection and diagnosis are of paramount importance. Low-level, intermittent parasitemia in the chronic indeterminate phase constitutes a public health hazard not only because of the potential for transmission through blood donation, or organ and bone marrow donation but also because of possible congenital transmission. Chronic infection can remain undetected during the reproductive life of women emigrated from endemic areas, putting their offspring at risk. Moreover, congenital transmission can be multi-generational, as a US-born infected mother of Hispanic descent who acquired the infection congenitally from her mother may potentially transmit *T. cruzi *to her offspring transplacentally. Health care providers are even less likely to suspect congenital Chagas disease in the offspring of a US-born Hispanic woman. Public health officials and health care providers should anticipate an increased prevalence and incidence of congenital Chagas disease within areas where Latin American ("Hispanic") populations are growing.

## Conclusions

Chagas disease has traditionally been considered as an important public health issue in Latin American countries. However, the epidemiology of Chagas disease has been changing in recent times mostly as a consequence of urbanization and international migration from Latin America. Chagas disease constitutes an important public health threat in terms of morbidity and mortality in areas in the United States where immigrant populations from Latin America are significant. Awareness of the actual prevalence of *T. cruzi *infection in the immigrants from Latin America is of paramount importance. Estimates of *T. cruzi *antibody prevalence in the blood donor population in the United States [7,8,10-12] most likely do not reflect the true seroprevalence rates in the Latin American immigrant population, as discussed above. Current estimates of the prevalence of Chagas disease in the Hispanic immigrant communities living in the US are based on demographic data, and not on actual seroprevalence data obtained from testing on the immigrant populations at risk. Our study highlights the importance of testing for Chagas disease in the populations most at risk, and the need for current data on the actual seroprevalence in areas where such immigrant populations are growing. Larger-scale epidemiologic surveys on Chagas disease in the immigrant communities from Latin America are warranted.

## Competing interests

The authors declare that they have no competing interests.

## Authors' contributions

RA designed the study, obtained its IRB approval, performed the preliminary testing by ELISA, interpreted results, drafted and revised the manuscript for submission. CEM assisted with the medical chart review process and the data analysis. AYK and TEL provided technical knowledge for the ELISA reader and assisted with the reading for the preliminary testing by Hemagen method. PMS supervised the study including its design, IRB approval, preliminary and confirmatory testing, data analysis and interpretation, manuscript revision and submission. All authors read and approved the final manuscript.
